# MicroRNAs as markers of progression in cervical cancer: a systematic review

**DOI:** 10.1186/s12885-018-4590-4

**Published:** 2018-06-27

**Authors:** Barbara Pardini, Daniela De Maria, Antonio Francavilla, Cornelia Di Gaetano, Guglielmo Ronco, Alessio Naccarati

**Affiliations:** 1 Italian Institute for Genomic Medicine (IIGM), Via Nizza 52, 10126 Turin, Italy; 2 Department of Medical Sciences, University of Turin, Via Santena 19, 10126 Turin, Italy; 3 Center for Cancer Epidemiology and Prevention, AO City of Health and Science, Via Cavour, 31 10123 Turin, Italy

**Keywords:** Cervical cancer, Cancer progression, microRNA, Cervical intraepithelial neoplasia (CIN) lesions, HPV infection, Microarray, qPCR

## Abstract

**Background:**

Invasive cervical cancer (ICC) is caused by high-risk human papillomavirus types (HR-HPVs) and is usually preceded by a long phase of intraepithelial neoplasia (CIN). Before invasion, (epi) genetic changes, potentially applicable as molecular markers within cervical screening, occur in HPV host cells. Epigenetic alterations, such as dysregulation of microRNA (miRNA) expression, are frequently observed in ICC. The mechanisms and role of miRNA dysregulation in cervical carcinogenesis are still largely unknown.

**Methods:**

We provide an overview of the studies investigating miRNA expression in relation to ICC progression, highlighting their common outcomes and their weaknesses/strengths. To achieve this, we systematically searched through Pubmed database all articles between January 2010 and December 2017.

**Results:**

From the 24 studies retrieved, miR-29a and miR-21 are the most frequently down- and up-regulated in ICC progression, respectively. Microarray-based studies show a small overlap, with miR-10a, miR-20b, miR-9, miR-16 and miR-106 found repeatedly dysregulated. miR-34a, miR-125 and miR-375 were also found dysregulated in cervical exfoliated cells in relation to cancer progression.

**Conclusions:**

The pivotal role of miRNAs in ICC progression and initial development is becoming more and more relevant. Available studies are essentially based on convenience material, entailing possible selection bias, and frequently of small size: all these points still represent a limitation to a wide comprehension of miRNAs relevant for ICC. The targeted approach instead of a genome-wide investigation still precludes the identification of all the relevant miRNAs in the process. The implementation of deep sequencing on large scale population-based studies will help to discover and validate the relation between altered miRNA expression and CC progression for the identification of biomarkers. Optimally, once explored on a miRNome scale, small specific miRNA signatures maybe used in the context of screening.

**Electronic supplementary material:**

The online version of this article (10.1186/s12885-018-4590-4) contains supplementary material, which is available to authorized users.

## Summary

Altered microRNA expression is observed in cervical cancer and precancerous lesions. Reviewing the literature, miR-29a and miR-21 are frequently dysregulated in cancer progression. However, microarray-based studies show a small overlap, most studies are based on convenience material and frequently small.

## Background

Invasive cervical cancer (ICC) is the fourth most common cancer in women worldwide with an estimated 528,000 new cases and 266,000 deaths in 2012 [1]. Infection by Human Papillomavirus (HPV) is a necessary cause of ICC [2] but the large majority of infections clear spontaneously [3]. Persistent infections can result in intraepithelial lesions, commonly histologically classified as cervical intraepithelial neoplasia (CIN) grade 1 to 3. About 1/3 of CIN3 progress to invasion in 30 years [4]. CIN1 is considered as a morphologic expression of HPV infection and CIN2 as a mixture of CIN1 and CIN3, frequently regressing [5]. The mechanisms of carcinogenesis are only partly understood. Progression to intraepithelial pre-cancers involves the disruption of cell cycle control pathways mediated by overexpression of the viral E6 and E7 proteins that, among other actions, functionally inactivate the products of the RB and p53 immuno-suppressive genes. Progression to invasion involves the accumulation of genetic errors. The overall process is complex and includes a number of genetic and epigenetic alterations [6, 7].

Screening based on testing for HPV has been shown to be more effective than cytology-based ones and to allow longer screening intervals [8]. However, because of what described above, the positive predictive value of HPV testing for high-grade CIN is low, requiring further triage. Cytological triage is effective [8] but entails high frequency of colposcopy and test repeats [9]. Markers allowing to improve this process would be very useful, so as markers allowing to reduce the overtreatment of non-progressive CIN2/3.

miRNAs are short non-coding RNAs that modulate gene expression either by catalyzing mRNA cleavage or by inhibiting mRNA translation [10]. The mature miRNA is a single stranded ~ 22 nucleotide RNA, sequentially processed from a primary transcript (pri-miRNA) and the resulting stem-loop structure (pre-miRNA) by the Drosha and Dicer proteins, respectively [11]. miRNAs are epigenetic regulators. Many miRNAs are tissue- or differentiation-specific and their temporal expressions modulate gene expression by pairing with complementary nucleotide sequences of the target mRNAs [12]. miRNAs may be overexpressed or down regulated in cancers [13] and have been associated with genetic (e.g. deletions, amplifications and point mutations) and epigenetic (histone modifications and aberrant DNA methylation) alterations [14, 15]. Human miRNAs are frequently located at fragile sites and chromosomal regions affected in cancer. Therefore chromosomal alterations are thought to represent a major mechanism underlying altered miRNA expression in cancer, as already demonstrated in melanoma, neuroblastoma, myeloma cell lines and for ovarian and breast cancer [16].

Evidence from cell lines and (pre) malignant lesions supports the involvement of miRNAs at every stage of ICC development [17–21]. However, little is still known about the specific miRNAs involved and the mechanisms behind their deregulation [22]. Aberrant miRNA expression seems to result from HR-HPV infection [18]. Some miRNA loci localize to fragile sites, where HR-HPV DNA integration may occur. Proteins encoded by HR-HPV can influence host miRNAs expression. HR-HPV E6 and E7 proteins modulate the expression of DNA methyltransferases, enzymes regulating gene expression by methylating their promoter regions [22, 23].

The aim of this review is to comprehensively evaluate the published literature focused on miRNA profiling in relation to progression to ICC. We are aware that some of the studies hereby reported have been recently reviewed by others [24–26]. However, we have updated the studies and we have provided additional information not previously covered. In particular, we tried to identify common miRNA profiles (up- or down-regulated miRNAs) that occur during the progression from normal cervical epithelium, via different CIN lesions, to SCCs in primary tissue or in cervical scraping. The downstream effect on target genes of the identified miRNA signatures and relevant pathways emerging are also briefly explored. Finally, we also provide an overview on the experimental approaches so far used to analyze miRNA expression and the design of the studies, including information on available HPV genotyping.

## Methods

### Literature search

We selected all studies focused on the dysregulation of miRNA expression during progression of cervical carcinogenesis, especially in intraepithelial lesions. Between January 2010 and December 2017 we systematically searched PubMed for publications (non-review) in English initially with “miRNA/microRNA” and “cervical cancer” as keywords, then supplemented by “progression”, “CIN”, “pre-cancerous lesions”, “cervical exfoliated cells”. Besides this search, we searched literature in PubMed by using MeSH terms (Cervical Neoplasms and MicroRNAs) with the following approach: “Uterine Cervical Neoplasms” [MeSH Terms] AND (“microRNAs” [MeSH Terms] OR “microRNAs” [All Fields] OR “microRNA” [All Fields]) AND (“microRNAs” [MeSH Terms] OR “microRNAs” [All Fields] OR “miRNAs” [All Fields]) AND (“microRNAs” [MeSH Terms] OR “microRNAs” [All Fields] OR (“micro” [All Fields] AND “RNA” [All Fields]) OR “micro RNA” [All Fields]) AND (“microRNAs” [MeSH Terms] OR “microRNAs” [All Fields] OR “miRNA” [All Fields]) AND (“2006/01/01” [PDAT]: “2017/12/31” [PDAT]) AND Journal Article [ptyp] NOT Review [ptyp]. Two independent persons investigated the literature retrieved (A.N, D.D.M.).

Several studies were excluded according to the following criteria (one was sufficient for exclusion): (1) non-cervical cancers considered; (2) miRNAs searched in serum or plasma; (3) miRNAs studied only in cellular lines or in normal versus tumor tissue, without relation to progression from healthy to CIN lesions to SCC; (4) miRNAs investigated only in advanced disease, lymph node metastasis or in relation to prognosis or treatment, radiation and chemotherapy, or (5) studies based on functional experiments, miRNA–related polymorphisms, miRNA target genes, HPV or miRNA methylation, or reporting only computational approaches.

Twenty four studies were identified and included in the present review (See workflow of selection in Additional file 1: Figure S1).

For each study: author (s), publication year, number and characteristics of patients, analyzed tissue, HPV status and profiling platform and direction (up or down regulation) of differentially expressed miRNAs among different lesions were recorded. The PRISMA statement was followed for systematic reviews http://prisma-statement.org/PRISMAStatement/PRISMAStatement.aspx.

### miRNA target genes, gene enrichment and pathway analysis

For miRNAs reported as dysregulated in two or more studies (separately up- or down-regulated), we searched for validated target genes using the miRWalk 2.0 database (http://zmf.umm.uni-heidelberg.de/apps/zmf/mirwalk2) [27]. In the majority of situations the -3p or -5p miRNA arms was not specified in the retrieved studies, so we have arbitrarily selected one of them (if present) by an additional literature search on the most common arms explored in the cervical cancer field, or in cancer in general. Of all the retrieved validated target genes, we excluded those overlapping between up- and down-regulated miRNAs. Resulting lists of target genes were tested using the Enrichr software (http://amp.pharm.mssm.edu/Enrichr/), for their over-representation in biological pathways. Enrichr is an integrative web-based software application that includes gene-set libraries, available for analysis and download [28]. In particular, for the present research we investigated: KEGG (http://www.genome.jp/kegg/), and Virus Mint (http://amp.pharm.mssm.edu/Harmonizome/resource/Virus+MINT). The relevance of each gene set enrichment was assessed by a *p*-value adjusted for multiple testing based on the hypergeometric distribution. Gene sets with probability < 5% were considered as significantly overrepresented.

The final list of genes was also investigated in the Cervical Cancer Gene Database (CCDB) [29] which reports separately up- or down-regulated genes involved in ICC.

## Results

An overview of the 24 identified studies is reported in Table 1. Notably, some of them investigated the overall progression from normal epithelium to ICC, while others only focused on intraepithelial lesions. The techniques used most frequently to evaluate miRNA expression were i) quantitative real -time PCR (qPCR, 18 articles), based on candidate miRNA (s) [30–47], and ii) microarray, either by manufacturer or customized (five articles), [16, 48–51]. Only one study analyzed miRNA expression qualitatively by reverse-transcriptase PCR (RT-PCR) [52]. Array studies widely differed for the number of miRNAs investigated (from 202 to 875). In candidate-miRNA studies, authors analyzed only one or few miRNAs based on literature data or their own previous studies. A few studies performed a discovery phase (generally by arrays) with subsequent validation by a different technique (usually qPCR) [47, 49, 50], as recommended, for example, by the Minimum Information for Publication of Quantitative Real-Time PCR Experiments (MIQE) guidelines [53].

**Table 1:** Details and main outcomes on studies investigating miRNA expression in relation to CC progression

Ref.	Design of the study	Number of analysed miRNAs (technique)	Up-regulated miRNAs in CC progression			Down-regulated miRNAs in CC progression		
	Studies on biopies		SCC > CIN2/3 > CIN1/NORMAL	SCC > CIN2/3	CIN2/3 > CIN1/NORMAL	CIN1/NORMAL > CIN2/3	CIN2/3 > SCC	NORMAL/CIN 1 > CIN2/3 > SCC
Pereira PM et al., 2010 [48]	Samples (*n* = 25): 4 SCC, 5 HSIL, 9 LSIL and 19 normal cervical tissues snap-frozen in liquid nitrogen and stored -80C HPV genotyping not available (only HPV + or -)	281 miRNAs (in house microarray)	miR-10a miR-132 miR-148a miR-196a miR-302b	miR-16 miR-27a miR-197 miR-106a miR-142-5p miR-205			miR-522*(miR-522-5p) miR-512-3p	miR-26a miR-29a miR-99a miR-143 miR-145 miR-199a miR-203 miR-513
Li B et al., 2010 [30]	Samples (*n* = 140): 108 tissue biopsy of patients with abnormal cytological results and/or HR-HPV infection, 32 normal with HPV, 32 normal without HPV, 32 CIN with HPV, 12 CIN without HPV infection and 32 CC HPV genotyping not available (only HPV + or -)	1 miRNA (semi-quantitative RT-PCR)						pri-mir-34a
Li Y et al., 2010 [31]	Samples (*n* = 78): tissue biopsy of patients with 22 CIN1, 27 CIN2 and 29 CIN3 (washed in sterile saline and placed in RNA later and then stored in liquid nitrogen) HPV genotyping available	1 miRNA (qPCR)						miR-218
Li Y et al., 2011 [49]	Samples (*n* = 18): tissue biopsy of patients 6 HPV16-positive SCCs, 6 HPV 16-positive CIN2/3, and 6 normal cervical tissues for discovery Samples (*n* = 91): tissue biopsy of patients 24 HPV positive SCCs, 24 HPV positive CIN2/3 and 43 normal cervical tissues for validation Snap-frozen in liquid nitrogen and stored -80C HPV genotyping available	Discovery: 875 miRNAs (microarray) Validation: 6 miRNAs (qPCR)	miR-15b miR-16 miR-17 miR-20a miR-20b miR-25 miR-31 miR-92a (also by validation) miR-92b miR-93 miR-106a miR-182 miR-185 miR-155 (also by validation) miR-221 miR-222 miR-224					let-7b miR-10b miR-29a (also by validation) miR-29c miR-99a (also by validation) miR-100 miR-125b miR-126 miR-145 miR-195 (also by validation) miR-199a-3p miR-218 miR-375 (also by validation) miR-424
Li BH et al., 2011 [32]	Samples (*n* = 125): 20 negative HR-HPV normal cervical epithelium, 20 HR-HPV infected normal cervical epithelium, 14 CIN1, 13 CIN2, 16 CIN3 and 42 CC Snap frozen in liquid nitrogen and stored -70C HPV genotyping not available	1 miRNA (qPCR)					miR-100	
Deftereos G et, 2011 [33]	Samples (*n* = 142): 23 normal histology and negative HR- HPV, 21 HR-HPV, 15 CIN1, 51 CIN2/3 (CIS), 23 ICC Samples were FFPE HPV genotyping available (only HPV + or -)	2 miRNAs (qPCR)	miR-21					
Cheung TH et al., 2012 [51]	Samples (n = 33): biopsy specimens of patients with 12 CIN2, 12 CIN3, 9 CC and 9 normal epithelial cells for discovery. Samples (*n* = 24): 6 CIN2 and 18 CIN3 new and, 9 normal from discovery for validation All tissue specimens were embedded into Optimal cutting temperature compound and stored at − 80 °C	202 miRNAs (qPCR)	miR-9 miR-20b		miR-10a miR-34b miR-34c miR-338 miR-345 miR-424 miR-512-5p miR-518a			miR-193b miR-203
Wilting SM et al., 2013 [16]	Samples (*n* = 47): 10 SCC, 9 AdCAs, 18 CIN2/3 and 10 normal cervical squamous epithelial tissue No information on sample collection/storage HPV genotyping available	472 miRNAs (Agilent microarray)	let-7i miR-19b miR-21 miR-25 miR-28-5p miR-30e miR-34a miR-34b*(miR-34b-5p) miR-92a miR-92b miR-106b miR-146a miR-181d miR-200a*(miR-200a-5p) miR-206 miR-338-5p miR-592 miR-595	miR-7d miR-9 miR-15a miR-15b miR-16 miR-17 miR-17*(miR-17-3p) miR-18a miR-19a miR-20b miR-24 miR-27 miR-30d miR-93 miR-106a miR-107 miR-130b miR-141 miR-151-3p miR-155 miR-185 miR-200c miR-331-3p miR-339-5p miR-363 miR-425 miR-652	let-7 g miR-10a miR-26a miR-29a miR-29b miR-29c miR-30a miR-34c-5p miR-101 miR-125a-5p miR-135b miR-143 miR-145 miR-146b-5p miR-150 miR-181b miR-191 miR-192	miR-193a-3p miR-205 miR-212 miR-221 miR-27a miR-27b miR-484 miR-636 miR-770-5p	miR-100 miR-125b miR-148a miR-188-5p miR-195 miR-199a-5p miR-199b-3p miR-218 mir-26b miR-375 mir-376a miR-378 miR-486-5p miR-494 miR-497 miR-513b miR-660 miR-671-5p miR-99a	miR-134 miR-149 miR-193b miR-203 miR-210 miR-23b miR-296-5p miR-365 miR-370 miR-493 miR-572 miR-575 miR-617 miR-622 miR-638
Bierkens M et al., 2013 [52]	Samples (*n* = 28): frozen samples of 6 HPV-positive normal cervical squamous epithelial specimens, 13 CDKN2A-positive hg-CIN and 9 SCC HPV genotyping not available	1 miRNA (RT-PCR)						miR-375
Wang X et al., 2014 [34]	Samples (*n* = 158): 38 normal cervical tissues without HPV infection, 13 CIN1/2, 39 CIN3 and 68 CC tissues with HR-HPV infection HPV genotyping available	8 miRNAs (qPCR)	miR-7a miR-25 miR-92a miR-378 (miR-25, miR-92a, and miR-378 more elevated in HPVpositive group than in negative)	miR-16				miR-29a
Leung C et al., 2014 [35]	Samples (*n* = 37): micro-dissected cervical specimens from 10 SCC, 16 CIN2/3 and 11 CIN 1 HPV genotyping not available	1 miRNA (qPCR)	miR-135a					
Villegas-Ruiz V et al., 2014 [47]	Samples (*n* = 8): 4 healthy, 4 CC (and 12 cell lines) for discovery (but not on cancer progression) Samples (*n* = 45): 25 CC, 10 HSIL, 10 LSIL and healthy cervical tissue obtained from patients subjected to hysterectomy by uterine myomatosis for validation on cancer progression HPV typing and sequencing The biopsies were stored in RNA later	Discovery: 7788 miRNAs of different organisms (Affimetrix microarray): 942 different in tumor vs. healthy (518 up and 424 down) but only 123 humans Validation: 1 miRNA in relation to progression (qPCR)		miR-196a				
Zeng K et al., 2015 [50]	Samples (n = 12): 3 SCC, 3 HSIL (CIN2 or CIN3), 3 LSIL (CIN1) and 3 normal cervices for discovery Samples (*n* = 103): 40 SCC, 35 HSIL, 15 LSIL and 13 normal cervices for validation HPV genotyping available	Discovery: 866 human and 89 viral miRNAs (Agilent microarray) in tumor vs. healthy tissue Validation: 9 miRNAs in relation to progression (qPCR)	miR-9 miR-21 miR-31					miR-195 miR-199b-5p miR-218 miR-376a miR-497
Gocze K et al., 2015 [36]	Samples (*n* = 98): FFPE tissue samples of 38 SCC, 20 HPV-positive CIN3, 10 HPV-positive CIN2 and 30 HPV-positive CIN1 HPV genotyping not available	6 miRNAs (qPCR)	miR-21 (but not statistically significant) miR-27a miR-155		miR-196			miR-34a miR-203
Shishodia G et al., 2015 [37]	Samples (*n* = 102): fresh cervical tissue collected in PBS from 56 cancer lesions, 23 premalignant (and 23 non-malignant control tissues (comprising 3 normal and 20 tissues with cervicitis) HPV genotyping available	2 miRNAs (qPCR)	miR-21					let- 7a
Zheng W et al., 2015 [38]	Samples (n = 140): FFPE tissue samples of 80 SCC, 30 HSIL, 15 LSIL, 15 benign gynecological disease hysterectomy as controls HPV genotyping available only + or - for SCC	1 miRNA (qPCR)	miR-31 (but only SCC resulted significantly increased)					
Ma L et al., 2015 [39]	Samples (*n* = 163): FFPE tissues samples of 29 SCC, 99 CIN, 35 cervicitis HPV genotyping not available	1 miRNA (qPCR)	miR-146a					
Bumrungthai S et al., 2015 [40]	Samples for miRNA profiling (*n* = 111): Fresh tissue from 43 SCC, 22 CIN II-III, CIN I, 12 cervicitis and 20 normal HPV genotyping available, only + or -	1 miRNA (qPCR)	miR-21 (no significant increase between CIN II-III and CIN I)					
Jimenez-Vences H et al., 2016 [41]	Samples for miRNA profiling (*n* = 49): 16SCC, 16 LSIL, 7 NON-SIL HPV16 positive, 10 NON-SIL-HPV-negative HPV genotyping available (only HPV16 or negative in the study)	3 miRNAs (qPCR)	miR-193b				miR-218 miR-124 (No clear trend since these miRNAs were also higher than in NON-SIL samples)	
Wen F et al., 2017 [42]	Samples (*n* = 607): fresh tissue from 185 SCC, 148 CIN II-III, 124 CIN I, 150 normal HPV genotyping available only + or – for SCC	1 miRNA (qPCR)	miR-15b					
Sun P et al., 2017 [43]	Samples (*n* = 165): fresh tissue from 56 SCC, 60 CIN (not further classified), 49 benign uterine disease as controls HPV genotyping available only + or - for SCC	1 miRNA (qPCR)	miR-466					
	***Studies on exfoliated cells***							
Tian Q et al., 2014 [44]	Samples (*n* = 1021): cervical exfoliated cells from 833 HPV - positive women underwent cervical biopsy under colposcopy, among whom 392 presented with abnormal pap test (ASCUS +) and 441 presented with normal Pap test but positive colposcopies, 188 with normal Pap tests and negative colposcopies did not undergo biopsies HPV genotyping available	6 miRNAs, selected from Li Y et al., 2011 (qPCR)						miR-34a miR-218 miR-375 miR-424
Ribeiro J et al., 2015 [45]	Samples (*n* = 114): cervical exfoliated cells (normal epithelium with HPV infection (20) and without (29), 28 LSIL, 29 HSIL and 8 ICC) HPV genotyping available	2 miRNAs (qPCR)						miR-34a miR-125b
Malta M et al., 2015 [46]	Samples (*n* = 73): cervical exfoliated cells (normal epithelium with (17) and without (21) HPV infection, 14 LSIL, 21HSIL) HPV genotyping not available	1 miRNA (qPCR)						let-7c

AdCA, adenomatous carcinoma, CIS, carcinoma in situ, ICC, invasive cervical cancer, SCC, squamous cervical cancer, HSIL, high-grade squamous intraepithelial lesion LSIL, low-grade squamous intraepithelial lesion, FFPE, formalin-fixed paraffin-embedded

In general, a great variability between studies can be observed, both as for the number of investigated subjects/samples (12 to 1021, most coming from convenience material without specification of selection methods) and the type of samples. Most studies analyzed formalin-fixed, paraffin-embedded tissue (FFPE) or frozen (stored in RNA later or PBS) cervical tissues. Only three studies used cervical exfoliated cells [44–46]. All specimens were collected at enrollment, before any treatment. Several studies reported HPV genotyping data or at least a stratification of samples in HPV-negative and positive but a few did not report any information.

The studies based on microarrays are first described and, within each, results are compared with those of ‘validation’ studies by qPCR conducted on the same miRNAs by the same or by other authors.

The first microarray study was published by Pereira and colleagues in 2010 [48]. Authors reported data obtained by an array spotted in house on 281 human miRNAs in 25 independent biological samples. Authors report high variability of miRNA expression, especially among normal samples and could not identify miRNAs significantly up- or down regulated in pre- or malignant vs. normal samples. In order to minimize such variability, authors prepared a pool of normal samples. With such approach they identified 21 miRNAs with statistically significant differential expression between the pool of normal samples, a group of CIN1 and CIN3 and the SCC samples. The expression of 8 of such miRNAs (miR-26a, miR-29a, miR-143, miR-145, miR-99a, miR-199a, miR-203, and miR-513) progressively decreased and that of 5 miRNAs (miR-10a, miR-132, miR-148a, miR-196a, andmiR-302b) progressively increased in these three groups. Conversely, there was a decrease moving from normal tissue to CIN followed by an increase from CIN to SCC for six miRNAs (miR-16, miR-27a, miR-106a, miR-142-5p, miR-197, and miR-205) and an increase followed by a decrease in two (miR-522*(now miR-522-5p) and miR-512-3p). No validation of the whole results was reported.

A subsequent array study was performed by Li Y and colleagues on 18 tissue samples, including HPV16-positive SSCs and CIN [49]. Out of 875 tested miRNAs, 31 (14 down-regulated and 17 up-regulated) showed significant trends from normal epithelium to cancer. Six of them (miR-29a, miR-92a, miR-99a, miR-155, miR-195, and miR-375) were validated and confirmed by qPCR in 91 biopsies (24 SCCs and 24 CIN2/3, 43 normal tissue). In particular, the down-regulation of miR-99a and miR-29a confirmed the results of Pereira et al. [48] just reported above. miR-218 was the most significantly down-regulated, as confirmed by the subsequent studies of Wilting et al. [16], Zeng et al. [50] discussed below and more recently by Jimenez-Vences [41]. In general, miR-218 was under-expressed in tissues infected by High Risk-HPV (HR-HPV) and more in CIN2/3 than in CIN1. In another study by Li Y and colleagues (same first name author, but a different group from that of the array study) [31], this miRNA also presented lower expression levels in patients with CIN2 and CIN3 than in those with CIN1 (78 CIN patients in total). This miRNA has hundreds of target genes, including *LAMB3,* which has been recognized to increase cell migration and to promote carcinogenesis in mouse models and in human keratinocytes [31]. Li Y and colleagues (of the array study [49]), also compared miRNA expression in CIN2/3 and SCCs with HPV16 infection to normal cervical tissues without HPV infection. The two groups showed significant difference only for miR-375 and miR-99a. Decreasing levels of miR-375, measured by qPCR, with more severe histology were observed also by Bierkens and colleagues on HPV-positive frozen biopsies (6 normal cervical squamous epithelial specimens, 13 *CDKN2A*-positive HG-CIN, and 9 SCCs) [52].

Finally, in the array study of Li Y and colleagues [49], miR-100 levels were significantly lower in CIN3 than in CIN1 and CIN2, as confirmed subsequently [16, 32]. This result was also validated in vitro by Li BH et al. in cervical cell lines [32]. Down-regulation of miR-100 by its specific inhibitor distinctly promoted cell growth, decreased cell apoptosis, and accelerated G2/M phase progression in HaCaT cells that constitutively express high level of this miRNA. These findings together suggest that a reduced miR-100 expression may contribute to cervical carcinogenesis by regulating cell growth, cell cycle and apoptosis.

In a study by Cheung and colleagues, the expression of a panel of 202 miRNAs was investigated in normal epithelium, CIN2, CIN3 and ICC samples using a qPCR platform [51]. Twelve miRNAs (10 up- and 2 down-regulated) were differentially expressed in CIN2/3 biopsies compared to normal cervical epithelial cells. This 12-miRNA signature could clearly separate CIN2/3 from normal tissue in an independent group of samples (6 CIN2, 18 CIN3, 9 normal) and patients with SCC (51) from individuals with normal cervical epithelium but not CIN2 from CIN3. Overall, miR-20b and miR-9 showed the highest fold change up-regulation. miR-9 expression was also significantly higher in SCC than in CIN2/3. Authors considered miR-9 of particular interest because it stimulates angiogenesis in a cell-type and context-dependent manner and it is up-regulated in several cancers [51], including ICC [48, 50]. A miR-9-mediated down-regulation of E-cadherin has been shown to lead to activation of β-catenin, resulting in the up-regulation of the target gene *VEGFA*, a proangiogenic factor [54]. Down-regulation of E-cadherin has been observed in both CIN and SCC and this is consistent with the progressive up-regulation of miR-9 in CIN and SCC described above [51].

Wilting and colleagues [16] investigated by a microarray the expression of 472 human miRNAs in 47 subjects (normal epithelium, CIN2/3, SCCs) and correlated their differential expression to histology. In total, 106 miRNAs were differentially expressed in CIN2/3 and/or SCCs compared with normal epithelium. Authors identified *early transient* miRNAs (*n* = 27; with significantly different expression in CIN2/3 compared to normal epithelia but with no difference in SCCs vs. normal), *late* miRNAs (*n* = 46; miRNAs having differential expression in SCCs compared with normal tissue and CIN2/3, but not in CIN2/3 compared with normal tissue), and *early continuous* miRNAs (*n* = 33; those molecules showing concordant differential expression in SCCs and CIN2/3 compared with normal) (details in Table 1). Some of the altered miRNA expression levels confirmed the results by Pereira et al. [48] and by Cheung et al. [51] (for instance miR-10a up-regulation and miR-203 down-regulation) and by a more recent study of Gocze and colleagues [36]. The latter group also confirmed Wilting` s observations of an increase of miR-155 in the transition from CIN1 to SCC, so as Li et al. [16, 49].

More recently, Zeng and colleagues [50] compared miRNA expression profiles in ICC, CIN and normal cervical tissues by microarray analysis and found several miRNAs as significantly dysregulated. In particular, 16 miRNAs were up-regulated and 10 were significantly down-regulated when comparing SCC to normal tissue. miR-21, miR-21-3p (formerly miR-21*), miR-15b and miR-16 were the most over-expressed while miR-218 and miR-376 were the most down-regulated. Forty four miRNAs were differentially expressed (13 up- and 31 down-regulated) when CIN2/3 were compared to normal cervical tissue. No significantly differentially expressed miRNAs were found when comparing CIN1 to normal tissue. Nine miRNAs (miR-21, miR-218, miR-376a, miR-31, miR-630, miR-9, miR-195, miR-497, and miR-199b-5p) differentially expressed in cervical samples with and without lesions were validated by qPCR in 103 samples with similar characteristics. miR-21, miR-31 and miR-9 were again significantly up-regulated in ICC, as found in other works reported in the present review [16, 33, 36, 37, 49, 51]. Among the other validated results, miR-218, miR-195, miR-497 and miR-199b-5p were significantly down-regulated in ICC and CIN2/3, while miR-376a was significantly down-regulated only in ICC but not in CIN2/3. However, they did not observe differential miRNA expression between CIN1 and normal tissue, Authors hypothesized that miRNAs down-regulated in both SCC and CIN2/3 may be involved in the abnormal transformation from pre-cancerous lesions to cancer.

Other studies investigated one or few miRNAs previously identified as dysregulated in ICC progression. Villegas-Ruiz and colleagues [47] after an initial investigation of miRNA profiles in tumor vs. healthy tissue by arrays, focused on the expression of miR-196a in relation to ICC progression. They showed increasing expression of this miRNA from healthy tissues to low grade and to high grade CIN and tumor, confirming the findings of Pereira et al. In the study of Gocze [36], miR-196a also showed an increasing expression from CIN1 to CIN2/3 but was significantly lower in SCC than in CIN2/3.

Deftereos et al. [33] analyzed miR-143 and miR-21 in a large set of samples with different stages of disease. Despite increasing down-regulation of miR-143 with increasing severity of histology had been previously reported [47, 48], such pattern was not confirmed in this study [33]. miR-21 expression had been observed to increase with more severe diagnosis in neoplasms of many sites. An overexpressed miR-21 was also found in cervical pre-cancerous lesions and in ICC [16, 36, 37, 40]. In the work of Deftereos et al. [33] its levels increased from low-to high-grade CIN and were highest in invasive cancer tissues. miR-21 was also analyzed by Shishoida and colleagues together with let-7a [37]. Increasing miR-21 expression levels were found associated with disease progression while lower levels of let-7a were detected in invasive cancer tissues compared to pre-cancerous lesions and normal control tissues. Among all tissue types, the highest levels of let-7a were detected in pre-cancerous lesions. Authors observed that, in the cervix, altered miR-21 and let-7a levels were strongly associated with aberrant expression and activation of STAT3 and might be closely linked with HPV16 infection. In this sense, miRNAs could mediate functional links between STAT3 and other transcription factors including, NF-kB. NF-kB pathway is an important player in the development of cervical cancer and with STAT3 cooperatively regulates a number of target genes including antiapoptotic and cell cycle control genes, and genes encoding for cytokines and chemokine [55].

Leung and colleagues observed an overexpression of miR-135a in SCC compared to CIN1, and CIN2/3 in FFPE samples [35]. Authors found that miR-135a regulates Wnt/β-catenin signaling through its target genes *SIAH1* and possibly *APC*. This may eventually cause transformation into ICC, with miR-135a also promoting the migration, invasion and proliferation abilities of cancer cells.

In the work of Wang et al. [34], increased levels of miR-25, miR-92a and miR-378 were observed in HPV-infected tissue groups in comparison with HPV negative tissues. These results were consistent with those obtained from HPV-infected raft tissues. Another miRNA, miR-27a, increased its expression from normal cervical tissues to CIN1,2,3 and ICC, in agreement with the study of Gocze et colleagues [36] but at variance with that of Wilting et al. [16], in which miR-27a was down-regulated when moving from normal tissue to ICC. MiR-27a is an oncogenic miRNA modulated by p53, E2F and c-Myc. Both HR-HPV E6 and E7 proteins interact with c-Myc by increasing its transcriptional activity [56]. Finally, in agreement with previous observations [49], miR-29a expression decreased from normal tissue to CIN and ICC.

The study of Li B. et al. [30] examined the expression of pri-miR-34a, the precursor of miR-34a, in normal cervical epithelium, CIN and ICC by semi-quantitative RT-PCR. The pri-miR-34a expression was significantly decreased in the CIN and ICC groups in comparison with normal cervical epithelium group as a function of grade. The reduction of pri-miR-34a expression associated with HR-HPV infection occurred before morphologic abnormalities of cervical epithelium and before the changes induced by HR-HPV E6 in the p53-dependent pathway. Thus, authors concluded that it is plausibly an early-onset event in ICC development and that miR-34a and its precursor can likely be regarded as potential molecular markers for cervical screening and molecular targets for blocking ICC development.

### miRNA alterations in ICC progression analyzed in exfoliated cells

Cervical exfoliated cells are widely used in ICC screening, both for HPV testing and Pap test. Recently, their use has been extended to miRNA analyses. To our knowledge, the first report on miRNA detection in cervical exfoliated cells was by Tian et al. in 2014 [44]. Six candidate miRNAs, selected among those identified by Li Y and colleagues [49], were investigated in samples from 1021 HPV-positive women. The levels of miR-218, miR-34a, miR-424 and miR-375 significantly decreased with increasing severity.

Results partially confirm those previously obtained in cervical tissue. In particular, a decrease in the levels of miR-424 and miR-375 with higher-grade cytology was observed, similar to that seen when moving from CIN1 to CIN3 tissues [49].

Ribeiro et al. [45] evaluated miR-34a and miR-125b levels in cervical exfoliated cells of women with different histology (normal epithelium with and without HPV infection, Low-grade CIN, High-grade CIN and ICC). The study revealed an increased expression of miR-125b among women with HPV infection but normal epithelium and a significant progressive down-regulation with increasing severity of cervical lesions, reaching an 80% reduction in ICC. Women with normal cervix and HPV infection had also increased miR-34a expression levels. Despite no significant correlation with severity of cervical lesions, its expression increased in ICC. Significantly lower levels of miR-34a were detected also in CIN2/3 when compared with CIN1 and in SCC when compared with CIN2/3 (similarly to what observed in tissues by Gocze et al. [36]). It has been observed that miR-34a is directly regulated by p53 and that HR-HPV E6 induces its inhibition through p53 [36]. These observations confirm that miR-34a acts as a tumor suppressive miRNA in HPV-induced cervical transformation. This alteration in miR-34a expression is associated with the presence of a single or of multiple HR-HPV types.

let-7c levels were also evaluated by qPCR in cervical exfoliated cells from women with normal epithelium or with CIN (but no ICC) in the study by Malta and colleagues [46]. let-7c was progressively down-regulated in CIN of increasing grade. No previous investigations characterized let-7c expression during the progression of cervical lesions to cancer. let-7c has been reported as a probable target of p53 and its expression is induced in response to p53 activation [46].

These few studies shows that the potential application of miRNA detection in cervical exfoliated cells deserves further exploration, also as an additional option for triage of HPV-positive women in population-based screening.

### Commonly identified dysregulated miRNAs: their overlap among studies and target enrichment

**Fig. 1:**
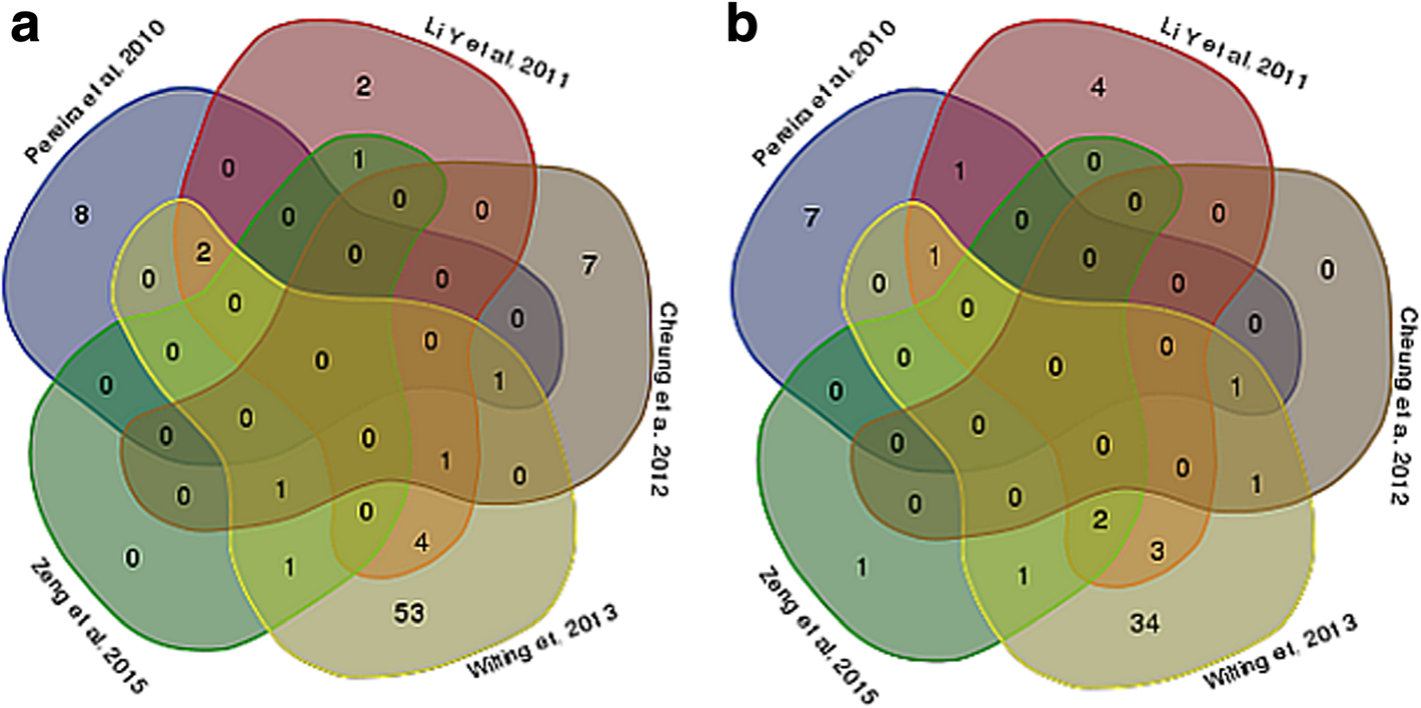
Venn diagram showing (A) up-regulated and (B) down-regulated miRNAs by studies that used microarrays to identify miRNAs in different stages of CC progression

The list of miRNAs found to be up- (17) or down- (13) regulated in relation to ICC progression and repeatedly observed among studies is reported in Table 2 and Additional file 2: Table S1. Among over expressed miRNAs, one (miR-21) was found to be associated to cervical carcinogenesis by five studies, nine (miR-9, miR-16, miR-25, miR-10a, miR-20b, miR-31 miR-92a, miR-106a and miR-155) by 3 studies and eight (miR-15b, miR-17, miR-27a, miR-92b, miR-93, miR-146a, miR-185 and miR-196a) by 2 studies. Among under-expressed miRNAs, for one (miR-218) an association was found by six studies, for two (miR-375 and miR-203) by four studies and for six (miR-99a, miR-29a, miR-195, miR-125b, miR-34a and miR-100) by three studies each. Fig. 1 reports the overlap between array-based studies, which was in fact very small.

**Table 2:** miRNAs up-regulated (A) and down-regulated (B) in CC progression identified in more than one study

References	Number of common miRNAs	miRNAs
A		
Li Y et al., 2011 [49]; Wilting et al., 2013 [16]	5	miR-15b^a^, miR-17, miR-92b, miR-93, miR-185
Li Y et al., 2011 [49]; Pereira et al., 2010 [48]; Wilting et al., 2013 [16]	2	miR-16, miR-106a
Li Y et al., 2011 [49]; Wang et al., 2014 [34]; Wilting et al., 2013 [16]	2	miR-92a, miR-25
Deftereos et al., 2011 [33]; Shishoida et al., 2015 [37]; Wilting et al., 2013 [16]; Zeng et al., 2015 [50]; Bumrungthai et al. 2015 [40]	1	miR-21
Cheung et al., 2012 [51]; Pereira et al., 2010 [48]; Wilting et al., 2013 [16]	1	miR-10a
Cheung et al., 2012 [51]; Li Y et al., 2011 [49]; Wilting et al., 2013 [16]	1	miR-20b
Gocze et al., 2015 [36]; Li Y et al., 2011 [49]; Wilting et al., 2013 [16]	1	miR-155
Cheung et al., 2012 [51]; Wilting et al., 2013 [16]; Zeng et al., 2015 [50]	1	miR-9
Li Y et al., 2011 [49]; Zeng et al., 2015 [50]; Zheng et al., 2015 [38]	1	miR-31
Gocze et al., 2015 [36]; Pereira et al., 2010 [48]	1	miR-27a
Wilting et al. 2015 [16]; Ma et al., 2015 [39]	1	miR-146a
Pereira et al., 2010 [48]; Villegas-Ruiz et al., 2014 [47]	1	miR-196a
B		
Li Y et al., 2011 [49]; Li Y et, 2010 [31]; Tian et al., 2014 [44]; Wilting et al., 2013 [16]; Zeng et al., 2015 [50]; Jimenez-Vences et al. 2016 [41]	1	miR-218
Bierkens et al., 2013 [52]; Li Y et al., 2011 [49]; Tian et al., 2014 [44]; Wilting et al., 2013 [16]	1	miR-375
Li Y et al., 2011 [49]; Pereira et al., 2010 [48]; Wilting et al., 2013 [16]	1	miR-99a
Li Y et al., 2011 [49]; Pereira et al., 2010 [48]; Wang et al., 2014 [34]	1	miR-29a
Cheung et al., 2012 [51]; Pereira et al., 2010 [48]; Wilting et al., 2013 [16]; Gocze et al. 2015 [36]	1	miR-203
Li Y et al., 2011 [49]; Wilting et al., 2013 [16]; Zeng et al., 2015 [50]	1	miR-195
Li Y et al., 2011 [49]; Ribeiro et al., 2015 [45]; Wilting et al., 2013 [16]	1	miR-125b
Gocze et al., 2015 [36]; Ribeiro et al., 2015 [45]; Tian et al., 2014 [44]	1	miR-34a
Li Y et al., 2011 [49]; Li. BH et al. 2011 [32]; Wilting et al., 2013 [16]	1	miR-100
Li Y et al., 2011 [49]; Pereira et al., 2010 [48]	1	miR-145
Li Y et al., 2011 [49]; Tian et al., 2014 [44]	1	miR-424
Cheung et al., 2012 [51]; Wilting et al., 2013 [16]	1	miR-193b^b^
Wilting et al., 2013 [16]; Zeng et al., 2015 [50]	1	miR-497

^a^miR-15b is also up-regulated in Wen et al. 2017 [42]

^b^miR-193b is up-regulated in Jimenez-Vences et al. 2016 [41]

The number of experimentally validated target genes for all the above mentioned miRNAs, retrieved in the miRWalk 2.0 database, is reported in Additional file 2: Table S2. Some 16,671 target genes were identified for the up-regulated miRNAs and 7075 for those down-regulated. After removing multiple miRNA binding sites for each target gene, genes overlapping within each group of miRNAs and those in common between up-regulated and down-regulated miRNAs, 3395 and 1575 genes, respectively, remained. Gene enrichment analysis for the final list of target genes of down-regulated miRNAs provided statistically significant over-representation and biologically plausible Kegg pathways terms (Additional file 2: Table S3). Subsequently, the enrichment analysis was focused only on the genes that were targeted by miRNAs found up-regulated in more studies (Additional file 2: Table S4). In this case, an overrepresentation of Kegg pathway terms (Additional file 2: Table S5) and Virus Mint terms emerged (Additional file 2: Table S6).

Finally, both original lists of miRNA validated target genes were investigated also in the CCDB database, where 257 genes over- and 110 under-expressed in ICC are listed. Among those genes previously identified as targets for down-regulated miRNAs (thus expected to be over-expressed with progression to ICC), 22 (8.5%) were indeed reported as over-expressed in ICC in CCDB. On the other hand, among the target genes of up-regulated miRNAs (thus expected to be under-expressed with increasing severity), 34 (30.9%) were also among the genes listed as under-expressed in ICC (Additional file 2: Table S7). Enrichment analyses have been performed on these resulting genes (Additional file 2: Tables S8, S9).

## Discussion

**Fig. 2:**
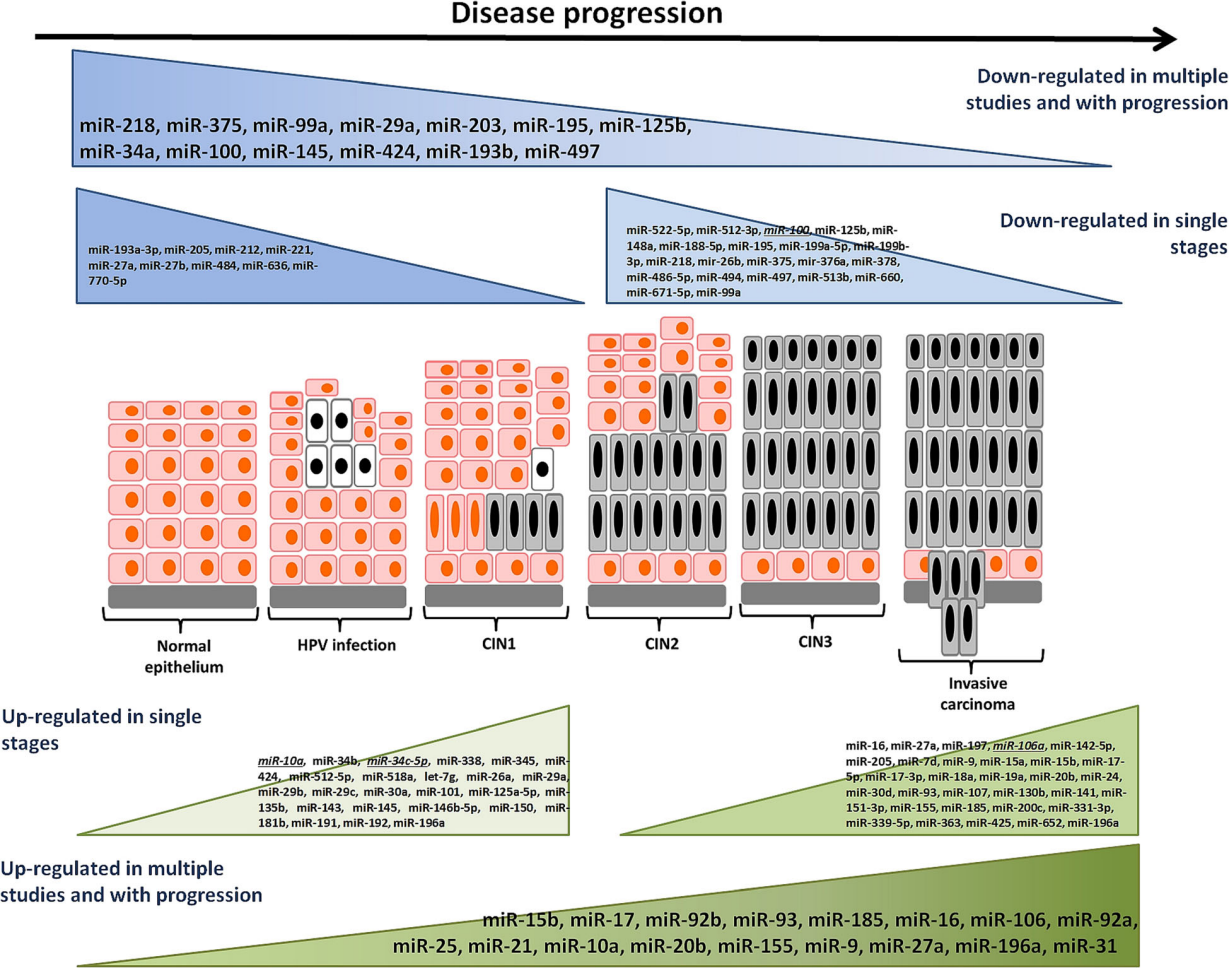
Summary of dysregulated miRNAs in the whole CC progression (miRNAs resulting from multiple studies) and miRNAs associated with progression in intermediate states of CC progression (miRNAs resulting from multiple studies are in Italics and underlined)

In summary, the expression levels of several miRNAs were repeatedly found to be associated to progression towards ICC via pre-cancerous lesions in different studies (Fig. 2). Interestingly, for many of them deregulation was not only confirmed by an internal validation in the same study but also with other techniques by other researchers. For instance, miR29a was found to be down-regulated with ICC progression [49] both when its expression levels were analyzed by an array-approach or tested by qPCR in an independent set of samples. Similar observations were obtained by Wang and colleagues [34], who first investigated the same miRNA by array and validated such results by qPCR on a large sample set.

Among up-regulated miRNAs, miR-21, which was found associated to CIN and ICC in 4 studies [16, 33, 37, 50], is of particular interest. miR-21 is one of the most abundantly expressed miRNA in mammals. Its up-regulation is associated with many cancers, both derived from solid tissues and of leukemic origin [57]. The identification of miR-21 function was the object of many research groups in the last 10 years. miR-21 interacts with a large number of targets and it is finely regulated in response to extracellular signals. Although miR-21 has been recognized as a key regulator of many processes involved in the control of cell survival and proliferation, it has also been linked to key processes in inflammation. Unlike other molecules, miR-21 is not simply characteristic of a pro-inflammatory or immunosuppressive status, but it acts as a key signal mediating their balance [57, 58]. Other miRNAs found altered in the present review, such as miR-125b, miR-155, and miR-146a, are known to be important in immune response and inflammation [59]. As an example, miR-125b is repeatedly observed down-regulated in relation to ICC progression. Interestingly, this miRNA has an important role in immune response and inflammation but can act as both an oncogene and oncosuppressor. miR-125b seems to be associated with HPV-induced carcinogenesis in two distinct pathways: a) it has homology for *HPV-L2*, which is needed for the viral capsid assembly (so it is possible that after early infection miR-125b can inhibit HPV replication and improve viral clearance); and b) miR-125b leads to p53-pathway inactivation, thus maintaining cells viable with viral genomes inside. This may result in the observed increased risk of HPV-genome integration and neoplastic lesion development [20]. To discern between an involvement of miRNAs in inflammatory processes related to HPV infection and their role in malignant transformation remains still an open issue, which needs to be furtherly explored [40].

Among down-regulated miRNAs, miR-34a, miR-125 and miR-375 were found to be progressively dysregulated when moving from normal epithelium to ICC, both in tissues and in exfoliated cells. This finding opens the possibility of an application in the screening process, to be tested in larger populations. From an epidemiological point of view, the main limitation of existing studies is the limited number of subjects included and, even more, in their being possibly selected. In principle, only samples including all lesions detected in a population-based screening and a random sample of healthy women from the same population (or of HPV-positive women without lesions if the conclusions are to be applied to HPV-positive women) are not subject to selection bias. Most studies seem, instead, to have been conducted on convenience material, whose origin and possible selection is usually not discussed in detail.

Some miRNAs were dysregulated in all stages of carcinogenesis, others only when moving from CIN2/3 to ICC [16, 36, 48, 51] (Fig. 2). In one study, miR-196 increased from normal epithelium to CIN3 and then decreases from CIN3 to ICC [36].

When investigating the role of the experimentally validated target genes of the miRNAs identified as dysregulated in cervical carcinogenesis, we found many pathways relevant to ICC onset and progression, such as signaling pathways. Interestingly, the overlap between the results of microarray-based studies was limited. This may be due to differences in study designs and populations (i.e. different ethnicities) or to the small number of ICC and CIN investigated. However, we must also report that all studies used different arrays, thus containing different number and type of miRNA probes or based on different array technology. The most interesting miRNAs found as dysregulated in multiple studies include miR-10a [16, 48, 51], miR-20b [16, 49, 51], miR-9 [16, 50, 51], miR-16 and miR-106a [16, 48, 49] (all up-regulated) and miR-99a [16, 48, 49], miR-203 [16, 48, 51], and miR-195 [16, 49, 50] (down-regulated).

Array-based analysis is restricted to miRNA molecules provided by databases and suffers from cross-detection-prone hybridization methods. As we could notice in the present review, despite the rapidly increasing number of miRNAs discovered and registered (currently 2656 human mature miRNAs; http://www.mirbase.org/index.shtml, Release 22 March 2018) the majority of studies focused on the first 800 miRNAs. Recently the use of next-generation sequencing (NGS) technology for miRNA expression profiling increased [60]. NGS offers a genome-wide approach and allows overcoming cross hybridization problems. Small RNA-sequencing, allowing also the detection of other similar small noncoding RNAs (such as piRNAs and tRNAs), has the greatest detection sensitivity and the highest accuracy in differential expression analysis. For the application of NGS techniques to small RNAs the standardization of experimental and computational protocols just begun [61]. Nevertheless, an important increase of studies applying NGS to miRNAs, also in cervical carcinogenesis, is expected in the next future [62]. The ideal approach would be to apply NGS on homogenous large scale studies for an hypothesis-free investigation of the whole miRNome to detect robust potential candidate (s) miRNA relevant for ICC progression as well as high-risk HPV infection. This is to avoid conflicting outcomes from small studies analyzing restricted lists of candidate miRNAs. Finally, small specific miRNA signatures should be evicted to be applied on a very extended scale, also in countries with more limited availability of funds for molecular analyses in the clinical routine. The first molecular characterization, including miRNAs, of 228 primary cervical cancers in comparison with healthy tissues has been recently described by The Cancer Genome Atlas (TCGA) network [63].

## Conclusions

In the context of ICC screening, an important issue to be investigated is whether miRNA signatures could be associated with current and future risk of HG-CIN, most likely in combination with other different markers, in order to improve risk stratification. Some of these markers could be used to identify very recently arisen HG-CIN. As the latter have very low probability of progression to invasion in short time and high probability of regression, this would allow a “wait and see” management, resulting in reduced overtreatment. The investigation of miRNA levels in cervical exfoliated cells certainly opens new possibilities for studying molecular markers in the context of screening programs.

## Additional files

Additional file 1: **Figure S1.** Workflow of selection of the studies included in the present Review. (TIF 1519 kb)

Additional file 2: **Table S1.** Dysregulated miRNAs in ICC progression in multiple studies as retrieved from the reviewed literature. **Table S2.** Validated target genes for the dysregulated miRNAs in ICC progression in multiple studies (data retrieved from miRWalk2.0). **Table S3.** Enrichment analysis for validated target genes of miRNA down-regulated in ICC progression (Kegg Pathways). **Table S4.** A group of 5 up-regulated miRNAs retrieved by multiple studies in the present review. **Table S5.** Enrichment analyses for genes targeted (*n*= 2620) by the above 5 miRNAs up-regulated in ICC progression (Kegg Pathways). **Table S6.** Enrichment analyses for genes targeted (*n*= 2620) by the above 5 miRNAs up-regulated in ICC progression (Virus Mint). **Table S7.** Altered expression levels of genes relevant for ICC (reported in CCDB) targeted by dysregulated miRNAs in ICC progression. (DOCX 39 kb)
