# Supplementary figures and images for: MicroRNAs as markers of progression in cervical cancer: a systematic review
Source: BMC Cancer. 2018 Jun 27;18:696. doi: 10.1186/s12885-018-4590-4 (PMC6020348; doi:10.1186/s12885-018-4590-4)

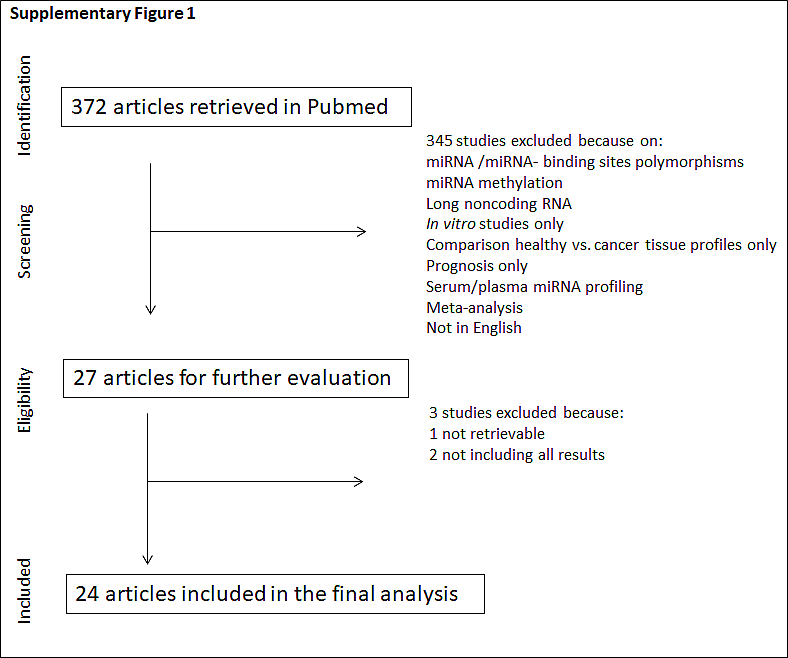

Supplement: Supplementary file 1 — Figure S1. Workflow of selection of the studies included in the present Review. (TIF 1519 kb) [file 12885_2018_4590_MOESM1_ESM.tif]
