# Supplementary material for: MicroRNAs as markers of progression in cervical cancer: a systematic review
Source: BMC Cancer. 2018 Jun 27;18:696. doi: 10.1186/s12885-018-4590-4 (PMC6020348; doi:10.1186/s12885-018-4590-4)
Supplement: Supplementary file 2 — Table S1. Dysregulated miRNAs in ICC progression in multiple studies as retrieved from the reviewed literature. Table S2. Validated target genes for the dysregulated miRNAs in ICC progression in multiple studies (data retrieved from miRWalk2.0). Table S3. Enrichment analysis for validated target genes of miRNA down-regulated in ICC progression (Kegg Pathways). Table S4. A group of 5 up-regulated miRNAs retrieved by multiple studies in the present review. Table S5. Enrichment analyses for genes targeted (n= 2620) by the above 5 miRNAs up-regulated in ICC progression (Kegg Pathways). Table S6. Enrichment analyses for genes targeted (n= 2620) by the above 5 miRNAs up-regulated in ICC progression (Virus Mint). Table S7. Altered expression levels of genes relevant for ICC (reported in CCDB) targeted by dysregulated miRNAs in ICC progression. (DOCX 39 kb) [file 12885_2018_4590_MOESM2_ESM.docx]

**Additional files**

**Table S1** Dysregulated miRNAs in ICC progression in multiple studies as retrieved from the reviewed literature

| **List of up-regulated miRNAs found in multiple studies** | |
| --- | --- |
| **As reported in the reviewed studies** | **As reported in miRBase** |
| miR-9 | hsa-miR-9-5p |
| miR-10a | hsa-miR-10a-5p |
| miR-15b | hsa-miR-15b-5p |
| miR-16 | hsa-miR-16-5p |
| miR-17 | hsa-miR-17-5p |
| miR-20b | hsa-miR-20b-5p |
| miR-21 | hsa-miR-21-5p |
| miR-25 | hsa-miR-25-3p |
| miR-27a | hsa-miR-27a-3p |
| miR-31 | hsa-miR-31-3p |
| miR-92a | hsa-miR-92a-3p |
| miR-92b | hsa-miR-92b-3p |
| miR-93 | hsa-miR-93-5p |
| miR-106a | hsa-miR-106a-5p |
| miR-146a | hsa-miR-146a-3p |
| miR-155 | hsa-miR-155-5p |
| miR-185 | hsa-miR-185-5p |
| miR-196a | hsa-miR-196a-5p |
| **List of down-regulated miRNAs found in multiple studies** | |
| **As reported in the reviewed studies** | **As reported in miRBase** |
| miR-29a | hsa-miR-29a-3p |
| miR-34a | hsa-miR-34a-5p |
| miR-99a | hsa-miR-99a-5p |
| miR-100 | hsa-miR-100-5p |
| miR-125b | hsa-miR-125b-5p |
| miR-145 | hsa-miR-145-5p |
| miR-193b | hsa-miR-193b-3p |
| miR-195 | hsa-miR-195-5p |
| miR-203 | hsa-miR-203 |
| miR-218 | hsa-miR-218-5p |
| miR-375 | hsa-miR-375 |
| miR-424 | hsa-miR-424-5p |
| miR-497 | hsa-miR-497-5p |

**Table S2** Validated target genes for the dysregulated miRNAs in ICC progression in multiple studies (data retrieved from miRWalk2.0)

| **miRNA down-regulated and all their target genes (n)** | **miRNA up-regulated and all their target genes (n)** |  | **miRNA down-regulated: target genes without multiple target sites (n)** | **miRNA up-regulated: target genes without multiple target sites (n)** |  | **miRNA down-regulated: unique target genes (no common targets with miRNA down-regulated) (n)** | **miRNA up-regulated: unique target genes (no common targets with miRNA up-regulated) (n)** |
| --- | --- | --- | --- | --- | --- | --- | --- |
| **7075** | **16671** |  | **3584** | **5987** |  | **1576** | **3564** |

**Table S3** Enrichment analysis for validated target genes of miRNA down-regulated in ICC progression (Kegg Pathways)

| **Index** | **Kegg Terms** | **P-value** | **Adjusted p-value** | **Z-score** | **Combined score** |
| --- | --- | --- | --- | --- | --- |
| 1 | Alcoholism_Homo sapiens_hsa05034 | 0.000008035 | 0.002210 | -1.90 | 22.33 |
| 2 | DNA replication_Homo sapiens_hsa03030 | 0.00003857 | 0.005304 | -1.94 | 19.67 |
| **3** | **Pathways in cancer_Homo sapiens_hsa05200** | **0.0003145** | **0.01730** | **-2.00** | **16.15** |
| 4 | Systemic lupus erythematosus_Homo sapiens_hsa05322 | 0.0001466 | 0.01344 | -1.70 | 14.98 |
| 5 | Cell cycle_Homo sapiens_hsa04110 | 0.0003042 | 0.01730 | -1.66 | 13.42 |
| **6** | **Viral carcinogenesis_Homo sapiens_hsa05203** | **0.001282** | **0.05036** | **-1.83** | **12.18** |
| 7 | Ras signaling pathway_Homo sapiens_hsa04014 | 0.001494 | 0.05136 | -1.83 | 11.89 |
| 8 | Non-alcoholic fatty liver disease (NAFLD)_Homo sapiens_hsa04932 | 0.001782 | 0.05444 | -1.76 | 11.12 |
| 9 | Metabolic pathways_Homo sapiens_hsa01100 | 0.002844 | 0.05532 | -1.69 | 9.90 |
| 10 | Hepatitis B_Homo sapiens_hsa05161 | 0.002621 | 0.05532 | -1.63 | 9.69 |

**Table S4** A group of 5 up-regulated miRNAs retrieved by multiple studies in the present review

| **miRNAs** |
| --- |
| hsa-mir-15b |
| hsa-miR-17-5p |
| hsa-miR-185-5p |
| hsa-miR-92b-3p |
| hsa-miR-93-5p |

**Table S5** Enrichment analyses for genes targeted (n= 2620) by the above 5 miRNAs up-regulated in ICC progression (Kegg Pathways)

| **Index** | **Kegg Terms** | **P-value** | **Adjusted p-value** | **Z-score** | **Combined score** |
| --- | --- | --- | --- | --- | --- |
| 1 | **Viral carcinogenesis_Homo sapiens_hsa05203** | 9.94E-12 | 2.82E-09 | -1.98 | 50.22 |
| 2 | **Pathways in cancer_Homo sapiens_hsa05200** | 9.51E-11 | 8.45E-09 | -2.06 | 47.45 |
| 3 | **Proteoglycans in cancer_Homo sapiens_hsa05205** | 5.83E-11 | 8.28E-09 | -1.99 | 46.83 |
| 4 | Hepatitis B_Homo sapiens_hsa05161 | 1.58E-10 | 8.45E-09 | -1.86 | 41.98 |
| 5 | HTLV-I infection_Homo sapiens_hsa05166 | 1.78E-10 | 8.45E-09 | -1.82 | 40.81 |
| 6 | Cell cycle_Homo sapiens_hsa04110 | 1.74E-10 | 8.45E-09 | -1.63 | 36.67 |
| 7 | Prostate cancer_Homo sapiens_hsa05215 | 3.60E-08 | 1.461E-06 | -1.8 | 30.93 |
| 8 | Endocytosis_Homo sapiens_hsa04144 | 5.14E-08 | 1.621E-06 | -1.77 | 29.63 |
| 9 | Chronic myeloid leukemia_Homo sapiens_hsa05220 | 5.13E-08 | 1.621E-06 | -1.71 | 28.68 |
| 10 | Epstein-Barr virus infection_Homo sapiens_hsa05169 | 6.12E-08 | 1.738E-06 | -1.73 | 28.66 |
| 11 | PI3K-Akt signaling pathway_Homo sapiens_hsa04151 | 2.15E-07 | 5.093E-06 | -1.78 | 27.35 |
| 12 | Insulin signaling pathway_Homo sapiens_hsa04910 | 9.68E-08 | 2.499E-06 | -1.53 | 24.65 |
| 13 | Neurotrophin signaling pathway_Homo sapiens_hsa04722 | 3.13E-07 | 6.055E-06 | -1.54 | 23.06 |
| 14 | Hippo signaling pathway_Homo sapiens_hsa04390 | 2.35E-07 | 5.123E-06 | -1.45 | 22.1 |
| 15 | Focal adhesion_Homo sapiens_hsa04510 | 3.84E-07 | 6.055E-06 | -1.49 | 22.08 |
| 16 | Pancreatic cancer_Homo sapiens_hsa05212 | 3.49E-07 | 6.055E-06 | -1.46 | 21.66 |
| 17 | Renal cell carcinoma_Homo sapiens_hsa05211 | 3.49E-07 | 6.055E-06 | -1.42 | 21.08 |
| 18 | MAPK signaling pathway_Homo sapiens_hsa04010 | 7.54E-07 | 9.836E-06 | -1.49 | 20.99 |
| 19 | RNA transport_Homo sapiens_hsa03013 | 4.85E-07 | 0.00000725 | -1.43 | 20.8 |
| 20 | Colorectal cancer_Homo sapiens_hsa05210 | 3.73E-07 | 6.055E-06 | -1.37 | 20.35 |

**Table S6.** Enrichment analyses for genes targeted (n= 2620) by the above 5 miRNAs up-regulated in ICC progression (Virus Mint)

| **Index** | **Virus Mint term** | **P-value** | **Adjusted p-value** | **Z-score** | **Combined score** |
| --- | --- | --- | --- | --- | --- |
| 1 | Epstein-Barr virus (strain GD1) | 8.24E-13 | 3.87E-11 | -1.19 | 33.12 |
| 2 | Human immunodeficiency virus 1 | 2.13E-08 | 5.01E-07 | -0.93 | 16.51 |
| 3 | Homo sapiens | 5.09E-07 | 7.977E-06 | -0.53 | 7.72 |
| 4 | **Human papillomavirus type 16** | 0.00003196 | 0.0003756 | -0.49 | 5.1 |
| 5 | Human adenovirus 5 | 0.0002571 | 0.002417 | -0.07 | 0.59 |
| 6 | **Human papillomavirus type 11** | 0.0007223 | 0.005658 | -0.03 | 0.21 |
| 7 | Human immunodeficiency virus type 1 (isolate 12) | 0.001115 | 0.007487 | 1.17 | -7.96 |
| 8 | Human adenovirus 2 | 0.002546 | 0.01496 | 1.46 | -8.72 |
| 9 | Simian virus 40 | 0.006243 | 0.0326 | 1.29 | -6.54 |
| 10 | Vaccinia virus (strain Western Reserve / WR) | 0.008744 | 0.03736 | 3.98 | -18.88 |
| 11 | **Human papillomavirus type 1a** | 0.01327 | 0.05196 | 3.01 | -12.99 |
| 12 | **Human papillomavirus type 18** | 0.0179 | 0.05608 | 2.93 | -11.8 |
| 13 | Human adenovirus E | 0.01716 | 0.05608 | 5.52 | -22.42 |
| 14 | Human herpesvirus 1 (strain 17) | 0.02658 | 0.07809 | 3.69 | -13.39 |
| 15 | **Human papillomavirus type 6b** | 0.03716 | 0.09704 | 4.57 | -15.03 |

**Table S7.** Altered expression levels of genes relevant for ICC (reported in CCDB) targeted by dysregulated miRNAs in ICC progression.

|  | |
| --- | --- |
| **Down-regulated miRNA target genes found in CCDB as over-expressed**  **(n=22)** | **Up-regulated miRNA target genes found in CCDB as down-regulated**  **(n=34)** |
| *APLP2* | *ANLN* |
| *CTSS* | *APOL2* |
| *DAP3* | *BCL2L1* |
| *EPB41L4B* | *CEACAM6* |
| *GARS* | *CRABP2* |
| *GATA3* | *CTGF* |
| *HYAL1* | *ERBB4* |
| *KLF3* | *FGFR2* |
| *KLK2* | *IER5* |
| *KRT10* | *IFI6* |
| *MEF2A* | *IMP3* |
| *NTN4* | *ITM2C* |
| *OAT* | *KIT* |
| *PA2G4* | *KRT7* |
| *PDIA3* | *KRT8* |
| *RTN3* | *MAP3K11* |
| *SERPINB5* | *MCM2* |
| *SMAD2* | *MMP1* |
| *TGFB1* | *MMP12* |
| *THBS1* | *MMP14* |
| *TIMP3* | *MYCN* |
|  | *NDRG1* |
|  | *NDUFS6* |
|  | *PCNA* |
|  | *PDGFRA* |
|  | *PIGF* |
|  | *PIGT* |
|  | *S100A8* |
|  | *SAA1* |
|  | *SFN* |
|  | *STAT1* |
|  | *TBC1D1* |
|  | *TPX2* |
|  | *VEGFC* |

| **Index** | **Kegg Terms** | **P-value** | **Adjusted p-value** | **Z-score** | **Combined score** |
| --- | --- | --- | --- | --- | --- |
| **1** | **Pathways in cancer_Homo sapiens_hsa05200** | **0.000003901** | **0.0002731** | **-2.11** | **26.28** |
| **2** | **Ras signaling pathway_Homo sapiens_hsa04014** | **0.00003834** | **0.001342** | **-1.98** | **20.16** |
| **3** | **PI3K-Akt signaling pathway_Homo sapiens_hsa04151** | **0.0002593** | **0.004538** | **-1.99** | **16.43** |
| 4 | Central carbon metabolism in cancer_Homo sapiens_hsa05230 | 0.0001996 | 0.004538 | -1.82 | 15.52 |
| 5 | Rap1 signaling pathway_Homo sapiens_hsa04015 | 0.0004357 | 0.006099 | -1.86 | 14.40 |
| 6 | Endocytosis_Homo sapiens_hsa04144 | 0.0009388 | 0.009388 | -1.81 | 12.65 |
| 7 | Cell cycle_Homo sapiens_hsa04110 | 0.001210 | 0.01058 | -1.56 | 10.45 |
| 8 | DNA replication_Homo sapiens_hsa03030 | 0.001704 | 0.01326 | -1.47 | 9.40 |
| 9 | Pancreatic cancer_Homo sapiens_hsa05212 | 0.005621 | 0.03935 | -1.68 | 8.70 |
| 10 | Glycosylphosphatidylinositol(GPI)-anchor biosynthesis_Homo sapiens_hsa00563 | 0.0008212 | 0.009388 | -1.20 | 8.54 |
| **11** | **MAPK signaling pathway_Homo sapiens_hsa04010** | **0.009158** | **0.05082** | **-1.74** | **8.18** |
| 12 | HTLV-I infection_Homo sapiens_hsa05166 | 0.009453 | 0.05082 | -1.67 | 7.78 |
| 13 | Prostate cancer_Homo sapiens_hsa05215 | 0.01001 | 0.05082 | -1.62 | 7.46 |
| 14 | AGE-RAGE signaling pathway in diabetic complications_Homo sapiens_hsa04933 | 0.01275 | 0.05951 | -1.66 | 7.25 |
| 15 | Cytokine-cytokine receptor interaction_Homo sapiens_hsa04060 | 0.01016 | 0.05082 | -1.56 | 7.18 |
| **16** | **TNF signaling pathway_Homo sapiens_hsa04668** | **0.01499** | **0.06560** | **-1.51** | **6.35** |
| 17 | Toxoplasmosis_Homo sapiens_hsa05145 | 0.01712 | 0.07050 | -1.49 | 6.04 |
| 18 | Hepatitis B_Homo sapiens_hsa05161 | 0.02549 | 0.09391 | -1.48 | 5.42 |
| 19 | Phospholipase D signaling pathway_Homo sapiens_hsa04072 | 0.02485 | 0.09391 | -1.46 | 5.41 |

**Table S8** Enrichment analyses for overexpressed genes in CCDB (Kegg Pathways)

| **Index** | **Kegg Terms** | **P-value** | **Adjusted p-value** | **Z-score** | **Combined score** |
| --- | --- | --- | --- | --- | --- |
| 1 | Inflammatory bowel disease (IBD)_Homo sapiens_hsa05321 | 0.00004828 | 0.001731 | -1.98 | 19.63 |
| **2** | **Proteoglycans in cancer_Homo sapiens_hsa05205** | **0.00006531** | **0.001731** | **-1.99** | **19.15** |
| **3** | **TGF-beta signaling pathway_Homo sapiens_hsa04350** | **0.0001039** | **0.001835** | **-1.81** | **16.64** |
| 4 | Malaria_Homo sapiens_hsa05144 | 0.001316 | 0.01744 | -1.82 | 12.09 |
| 5 | Colorectal cancer_Homo sapiens_hsa05210 | 0.002099 | 0.01825 | -1.85 | 11.39 |
| 6 | Pancreatic cancer_Homo sapiens_hsa05212 | 0.002374 | 0.01825 | -1.83 | 11.04 |
| 7 | Endocytosis_Homo sapiens_hsa04144 | 0.002755 | 0.01825 | -1.79 | 10.55 |
| **8** | **MicroRNAs in cancer_Homo sapiens_hsa05206** | **0.004050** | **0.02147** | **-1.69** | **9.32** |
| **9** | **p53 signaling pathway_Homo sapiens_hsa04115** | **0.002592** | **0.01825** | **-1.56** | **9.32** |
| 10 | AGE-RAGE signaling pathway in diabetic complications_Homo sapiens_hsa04933 | 0.005461 | 0.02553 | -1.78 | 9.25 |
| **11** | **Antigen processing and presentation_Homo sapiens_hsa04612** | **0.003215** | **0.01893** | **-1.53** | **8.79** |
| 12 | Chagas disease (American trypanosomiasis)_Homo sapiens_hsa05142 | 0.005781 | 0.02553 | -1.53 | 7.91 |
| **13** | **Lysosome_Homo sapiens_hsa04142** | **0.007996** | **0.03075** | **-1.47** | **7.11** |
| 14 | FoxO signaling pathway_Homo sapiens_hsa04068 | 0.009293 | 0.03284 | -1.47 | 6.88 |
| 15 | Cell cycle_Homo sapiens_hsa04110 | 0.008122 | 0.03075 | -1.40 | 6.76 |
| 16 | Hippo signaling pathway_Homo sapiens_hsa04390 | 0.01215 | 0.03835 | -1.38 | 6.07 |
| 17 | Phagosome_Homo sapiens_hsa04145 | 0.01230 | 0.03835 | -1.32 | 5.82 |
| 18 | Tuberculosis_Homo sapiens_hsa05152 | 0.01619 | 0.04767 | -1.39 | 5.73 |
| 19 | HTLV-I infection_Homo sapiens_hsa05166 | 0.03232 | 0.08156 | -1.44 | 4.95 |
| **20** | **Pathways in cancer_Homo sapiens_hsa05200** | **0.06994** | **0.1282** | **-1.42** | **3.77** |

**Table S9** Enrichment analyses for under expressed genes in CCDB (Kegg Pathways)
